# Myeloid GRK2 Regulates Obesity-Induced Endothelial Dysfunction by Modulating Inflammatory Responses in Perivascular Adipose Tissue

**DOI:** 10.3390/antiox9100953

**Published:** 2020-10-04

**Authors:** María González-Amor, Rocío Vila-Bedmar, Raquel Rodrigues-Díez, Rosa Moreno-Carriles, Alba C. Arcones, Marta Cruces-Sande, Mercedes Salaices, Federico Mayor, Ana M. Briones, Cristina Murga

**Affiliations:** 1 Departamento Farmacología, Facultad de Medicina, Universidad Autónoma de Madrid, Instituto de Investigación Hospital La Paz, 28029 Madrid, Spain; maria.gonzalezamor@uam.es (M.G.-A.); raquel.rodrigues@uam.es (R.R.-D.); mercedes.salaices@uam.es (M.S.); 2 Ciber de Enfermedades Cardiovasculares (CIBERCV), 28028 Madrid, Spain; aconcepcion@cbm.csic.es (A.C.A.); mcruces@cbm.csic.es (M.C.-S.); fmayor@cbm.csic.es (F.M.J.); 3 Departamento de Ciencias Básicas de la Salud, Facultad de Ciencias de la Salud, Universidad Rey Juan Carlos (URJC), 28022 Madrid, Spain; rocio.vila@urjc.es; 4 Servicio de Angiología y Cirugía Vascular, Hospital Universitario La Princesa, 28006 Madrid, Spain; rmorca@gmail.com; 5 Departamento de Biología Molecular and Centro de Biología Molecular Severo Ochoa (CBMSO) UAM-CSIC, 28049 Madrid, Spain; 6 Instituto de Investigación Sanitaria, Hospital Universitario La Princesa, 28006 Madrid, Spain

**Keywords:** perivascular adipose tissue (PVAT), G protein-coupled receptor kinase 2 (GRK2), tumor necrosis factor-α (TNFα), NADPH oxidase (Nox), endothelial dysfunction

## Abstract

Perivascular adipose tissue (PVAT) is increasingly being regarded as an important endocrine organ that directly impacts vessel function, structure, and contractility in obesity-associated diseases. We uncover here a role for myeloid G protein-coupled receptor kinase 2 (GRK2) in the modulation of PVAT-dependent vasodilation responses. GRK2 expression positively correlates with myeloid- (CD68) and lymphoid-specific (CD3, CD4, and CD8) markers and with leptin in PVAT from patients with abdominal aortic aneurysms. Using mice hemizygous for GRK2 in the myeloid lineage (LysM-GRK2^+/−^), we found that GRK2 deficiency in myeloid cells allows animals to preserve the endothelium-dependent acetylcholine or insulin-induced relaxation, which is otherwise impaired by PVAT, in arteries of animals fed a high fat diet (HFD). Downregulation of GRK2 in myeloid cells attenuates HFD-dependent infiltration of macrophages and T lymphocytes in PVAT, as well as the induction of tumor necrosis factor-α **(**TNFα) and NADPH oxidase (Nox)1 expression, whereas blocking TNFα or Nox pathways by pharmacological means can rescue the impaired vasodilator responses to insulin in arteries with PVAT from HFD-fed animals. Our results suggest that myeloid GRK2 could be a potential therapeutic target in the development of endothelial dysfunction induced by PVAT in the context of obesity.

## 1. Introduction

Obesity is a very prevalent condition defined by increased adiposity and metabolic dysfunction that correlates in humans and animal models of disease with hypertension and vascular alterations, such as endothelial dysfunction or structural and mechanical alterations. Endothelium is a key player in the control of vascular tone through the release of vasodilator factors, which vary depending on the different vascular beds. For example, in conductance vessels, endothelium releases mostly NO that modulate vasoconstrictor responses, as shown by the effects of endothelium removal or NO synthase inhibition [1,2]. Apart from the visceral and subcutaneous fat depots, numerous blood vessels are surrounded by a specific type of adipose tissue termed perivascular adipose tissue (PVAT). Increasing evidence demonstrates that PVAT acts as a secretory and endocrine organ with a direct impact on vessel function, structure, and contractility [3]. In addition to adipocytes, other cell types, such as macrophages, lymphocytes, and fibroblasts, are found in PVAT and may also contribute to its function. Moreover, when compared with other adipose tissue depots, PVAT shows a distinct pattern of expression of pro-inflammatory and adipokine mediators and has particular morphological features [4]. 

PVAT is able to secrete vasodilator but also vasoconstrictor molecules, and its overall net effect on vessels depends on multiple factors, such as the vascular bed, vessel type, species, and pathophysiological status [5]. In particular, in the setting of obesity, adipose depots undergo complex remodeling marked by adipocyte hypertrophy, an altered adipokine secretion pattern, increased infiltration of immune cells, and upregulation of pro-inflammatory cytokines, such as tumor necrosis factor-α (TNFα), among many others. An upregulation of TNFα expression has been detected in isolated adipocytes from PVAT of obese patients [6], but macrophages are also a major source of this cytokine [7]. In addition, TNFα has been found to induce chronic inflammation in PVAT of the abdominal aorta, which is related to the development of aortic aneurysms and other vascular complications [8]. Importantly, many of the PVAT-derived contractile factors are released by immune cells infiltrating this tissue [4], and, in isolated abdominal arteries from human obese patients, local inflammation through the production of TNFα and other cytokines abolishes some anti-contractile properties of PVAT [9,10]. In models of obesity and hypertension, PVAT serves to stimulate recruitment of monocytes and lymphocytes to arteries by enhancing chemokines and superoxide production in vascular cells [4]. In fact, PVAT from obese individuals exhibits increased recruitment of macrophages, oxidative stress, and inflammation, which leads to a loss of PVAT-mediated vasodilatory effects. So, in this condition, PVAT aggravates endothelial dysfunction, vascular remodeling, arterial stiffness, and atherosclerosis [5] and may also have a role in the development of aortic aneurysms [8]. This influence of PVAT in the vascular wall would add to the contribution of the endothelium and altered vascular contractility to the development of different types of aneurysms [11,12,13,14]. Numerous other mechanisms have been suggested to underlie the crosstalk between PVAT and vascular function, the regulation of PVAT inflammation being one of them. However, the actual trigger and the precise molecular dynamics of this process remain poorly understood.

G protein-coupled receptor kinase 2 (GRK2), a serine/threonine kinase that desensitizes multiple members of the G protein-coupled receptor (GPCRs) family, as well as other cellular signaling proteins [15,16], plays an important role in the control of adiposity and insulin sensitivity in physiological and pathological settings [15,16,17]. Particularly, GRK2 targeting is able to prevent and also to revert insulin resistance and excessive weight gain in different animal models of disease [15,18,19]. Downregulation of GRK2 decreases steatosis and fibrosis in cardiac tissue [20] and also prevents diet-induced hepatic insulin resistance and steatohepatitis [21]. 

Interestingly, GRK2 is highly expressed in various cell types of the immune system and the levels and activity of this kinase change in these cells under different pathological conditions (see Reference [15,16,22]). Moreover, it has been suggested that GRK2 may have a potential role in the onset or development of inflammatory disorders or in human pathologies with an inflammatory basis [16]. In this context, we recently reported that a reduction of GRK2 levels in myeloid cells prevents the development of glucose intolerance and hyperglycemia after a high fat diet (HFD) by downregulating a pro-inflammatory macrophage profile [23]. Moreover, conditioned media of macrophages that are hemizygous for GRK2 and treated with lipopolysaccharide (LPS) has a reduced pro-inflammatory effect on naïve macrophages. So, reducing GRK2 levels in myeloid cells is, per se, able to attenuate pro-inflammatory activation of macrophages and preserve physiological features of adipose and hepatic tissues in the face of a HFD [23].

Given the key role of macrophages in obesity-related vascular complications, we hypothesized that changes in GRK2 levels in myeloid cells may alter the inflammatory pattern of PVAT and thus impact the development of diet-induced vascular dysfunction. We show here that GRK2 levels correlate with markers of immune infiltration in the PVAT of patients with aortic aneurysms. In addition, using a mouse model of myeloid-specific GRK2 targeting, we show that decreasing GRK2 levels in myeloid cells limits diet-mediated upregulation of pro-inflammatory cytokines and nicotinamide adenine dinucleotide phosphate (NADPH) oxidase subunits in PVAT, leading to reduced obesity-induced loss of vasodilation and preserving vascular function.

## 2. Methods

### 2.1. Patients

We used aortic perivascular adipose tissue (PVAT) from patients with abdominal aortic aneurysm (AAA) that was obtained during open surgery. Patients were diagnosed of AAA by computed tomography angiography. Inclusion criteria included having symptomatic or asymptomatic AAA with transverse or antero-posterior diameter ≥5.5 cm in men (≥5 cm in women). Exclusion criteria included endovascular aortic reconstructive therapy, inflammatory aneurysm, active neoplastic conditions, human immunodeficiency virus (HIV) positive serology, and pregnancy. Clinical and demographic characteristics of the studied population are included in Table 1. PVAT was obtained from 42 patients in the Angiology and Vascular Surgery Unit of the Hospital Universitario La Princesa (Madrid). The study was carried out in accordance with the Declaration of Helsinki, and the protocol was approved by the Ethics Committee of the Hospital Universitario la Princesa (PI-825). Patients gave informed consent.

### 2.2. Animal Experimental Design

Experiments were performed on 5-month-old male control mice and mice with a reduction of around 50% in GRK2 levels in myeloid cells (*LysM-GRK2^+/^*^−^) [23]. In particular, transgenic male mice overexpressing a nuclear-localized Cre recombinase inserted into the first coding ATG of the lysozyme 2 gene (Lyz2) (B6.129P2-Lyz2tm1(cre)Ifo/J), obtained from Jackson Laboratories (Bar Harbor, ME, USA), were mated to floxed homozygous GRK2 (GRK2^f/f^) female mice. GRK2^f/Δ^LysM-Cre^−/−^ controls (referred to as control mice) and GRK2^f/Δ^LysM-Cre^+/−^ (referred to as LysM-GRK2^+/−^) offsprings were used and genotyped as described [23]. Animals were bred under controlled conditions at 22 ± 2 °C, with a relative humidity of 50 ± 10% in a 12h light/dark cycle with free access to food and water in the animal facility of the Centro de Biología Molecular Severo Ochoa (Madrid, Spain). At 8 weeks of age, mice were continued on a normal diet (ND) (2018S Harlan-Teklad, 12% calories from fat) or were fed a HFD (Envigo (formerly Harlan), TD.07011, 54.4% calories from fat) for 12 weeks. All animal experimentation procedures conformed to the European Guidelines for the Care and Use of Laboratory Animals (Directive 86/609) and were approved by the Ethical Committees for Animal Experimentation of our Institutions and the Comunidad de Madrid (PROEX 048/15).

### 2.3. Vascular Reactivity Studies

Aorta from control and LysM-GRK2^+/−^ mice were isolated, and abdominal and thoracic aortic segments of around 2 mm in length were left with PVAT (PVAT+) or cleaned (PVAT−) and mounted in a wire myograph in order to study vascular reactivity by isometric tension recording. After a 30-min equilibration period in oxygenated Krebs Henseleit Solution (KHS) (115 mM NaCl, 25 mM NaHCO_3_, 4.7 mM KCl, 1.2 mM MgSO_4_·7H_2_O, 2.5 mM CaCl_2_, 1.2 mM KH_2_PO_4_, 11.1 mM glucose, and 0.01 mM Na_2_EDTA) at 37 °C and pH 7.4, segments were stretched to their optimal lumen diameter for active tension development. This was determined based on the internal circumference/wall tension ratio of the segments by setting their internal circumference, Lo, to 90% of what the vessels would have if they were exposed to a passive tension equivalent to that produced by a transmural pressure of 100 mm Hg. Segments were washed with KHS and left to equilibrate for 30 min; then, maximum response of the segments was tested by an initial exposure to a high K^+^ solution (K^+^-KHS, 120 mmol/L). After an equilibration period, aortic segments were precontracted with phenylephrine at ~35% K^+^-KHS contraction in order to perform a concentration–response curve to increasing concentration of acetylcholine (1 nM–10 µM). After washing the arteries and a 30 min equilibration period, aortic segments were precontracted again, and a concentration-response curve to increasing concentration of insulin (10 nM–3 µM) was carried out. Then, arteries were washed until they reached their basal tone in order to perform a concentration-response curve to phenylephrine (1 nM–30 µM). Finally, a concentration-response curve to the NO donor dietylamine-NONOate (DEA-NO, 1 nM-10 µM) (Sigma-Aldrich, San Luis, MO, USA, Cat. No. D5431) was performed in phenylephrine precontracted arteries. Phenylephrine responses were stable and concentration response curves were performed after reaching a steady-state.

Some segments were incubated with the specific inhibitor of NADPH oxidase (NOX)1, NOXA1ds (10 µM) (Calbiochem-Merck, Darmstadt, Germany, Cat. No. 5327610001) or with an anti-TNFα antibody (InVivoMAb, Clone XT3.11, Cat. No. BE0058) (10 µg/mL) 1 h before the concentration-response curve to acetylcholine. 

Vasodilator responses were expressed as a percentage of the previous tone generated by phenylephrine. Vasoconstrictor responses were expressed in mN per mm of length for each segment.

### 2.4. RNA Analysis

PVAT from mice or humans was crushed with a polytron homogenizer in TRIzol (Life Technologies Inc., Carlsbad, CA, USA) to obtain total RNA. RNA was reverse-transcribed using NZY First-Strand cDNA Synthesis Kit (Nzytec, Lisbon, Portugal). Quantitative PCR (qPCR) was performed in 7500 Fast ABI System (Life Technologies Inc.), using the mice and human assay sequences detailed in Table 2.

For Sybr green assays, PCR cycles proceeded as follows: initial denaturation for 30 s at 95 °C, followed by 40 cycles at 95 °C for 5 s and 60 °C for 30 s. Melting curve analysis was performed to show PCR product specificity. For Taqman assays, multiplex PCR was done following the default setting running program. To calculate the relative index of gene expression, we employed the 2^−ΔΔCT^ method, where β2-microglobulin and β-Actin served as the internal control for mice and human respectively when using Sybr green assays, and 18s served as the internal control for both mice and human samples when using Taqman assays. Samples from control mice fed a normal diet were used as calibrator where appropriate. 

### 2.5. Statistical Analysis 

All data are expressed as mean values ± standard mean error and n represents the number of animals or patients studied. Data are reported as dot plots that represent different biological replicates. When this is not possible, the number of animals is reported in figure legends. Statistical analysis was done by GraphPad Prism Software San Diego, CA, USA (v7.04). Data distribution (by Shapiro–Wilk normality test) was used to choose the appropriate statistical test. Results were analyzed by the Mann–Whitney non-parametric or Student’s *t*-tests when appropriate (two-tailed) or two-way ANOVA followed by a Tukey´s or Sidak´s multiple comparison tests. The exclusion of data from the analysis was done by the ROUT method with GraphPad Prism Software. Statistical analysis for the human PVAT study was also performed by GraphPad Prism. Univariate association was performed by Spearman correlation test. A *p* < 0.05 was considered significant.

## 3. Results

### 3.1. GRK2 Expression Positively Correlates with Myeloid and Lymphoid Markers and Leptin in Perivascular Adipose Tissue from Patients with Abdominal Aortic Aneurysm 

It has been suggested that PVAT might have a role in vascular damage particularly in obesity, at least in part because of the infiltration of inflammatory macrophages [8]. Moreover, PVAT is the major site for macrophage and T cell accumulation in human abdominal aortic aneurysm (AAA [24]). We used aortic PVAT from patients with AAA as a human model to analyze a potential correlation between GRK2 expression and that of myeloid or lymphoid immune cell markers in this specific adipose tissue depot in a situation of vascular damage.

**Figure 1:**
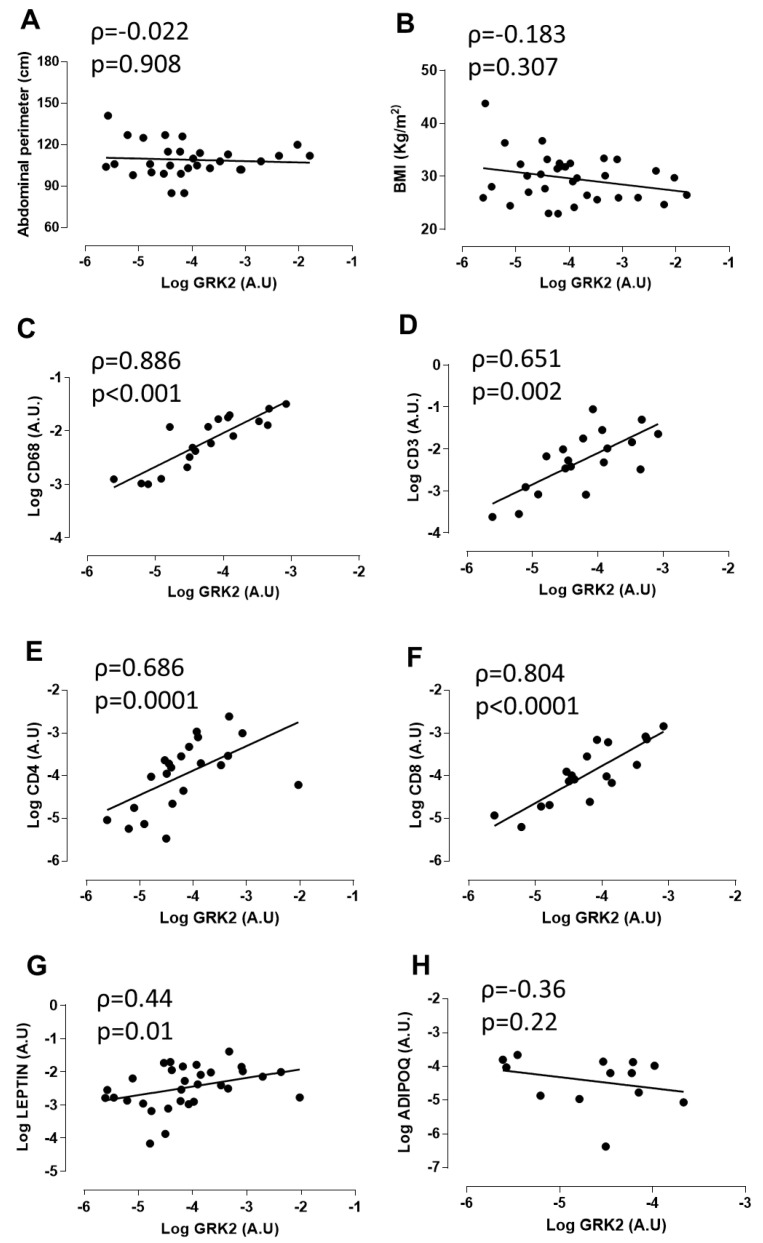
Correlation between abdominal perimeter (**A**), body mass index (BMI) (**B**), the macrophage marker CD68 (**C**), the T lymphocyte markers CD3 (**D**), CD4 (**E**), and CD8 (**F**), leptin (**G**), and adiponectin (**H**) and G protein-coupled receptor kinase 2 (GRK2) mRNA in human aortic perivascular adipose tissue. Univariate association was performed by Spearman correlation test. AU: arbitrary units.

These patients showed overweight according to their body mass index (BMI) and had central obesity with a mean abdominal perimeter of 109 ± 2.01 cm (see Table 1 for more information). As shown in Figure 1A,B, the total mRNA levels for GRK2 in PVAT did not correlate with abdominal perimeter or with BMI. However, there was a positive and highly significant correlation between GRK2 expression in PVAT and that of the macrophage marker CD68 (Figure 1C). We also detected a clear and statistically significant correlation between GRK2 expression in PVAT from these patients and that of specific markers of T lymphocytes, such as CD3, CD4, and CD8 (Figure 1D–F). In addition, GRK2 expression positively correlated with leptin levels (1G). However, no correlation was observed with adiponectin (1H). Altogether, these data indicated that the positive relationship between GRK2 expression and immune cell infiltration in PVAT is not merely a consequence of enhanced/altered body weight in human patients with vascular damage and were consistent with our hypothesis that GRK2 expression in myeloid cells may modulate the inflammatory and immune landscape of PVAT and, thus, vascular damage.

### 3.2. GRK2 Downregulation in Myeloid Cells Preserves Endothelium-Dependent Relaxation in Arteries with PVAT from Obese Animals

**Figure 2:**
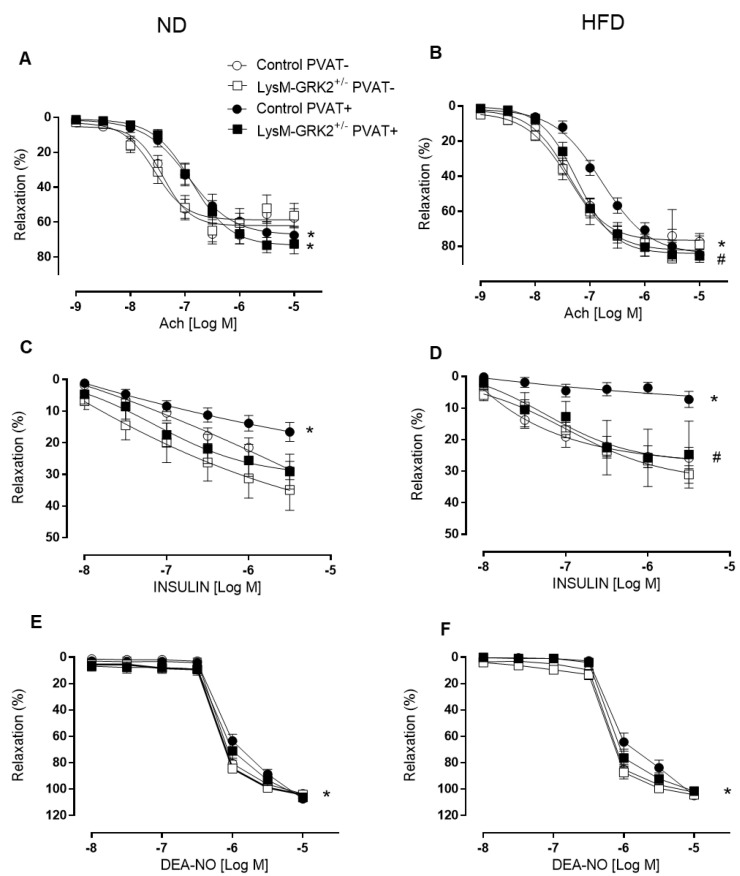
Concentration-response curves to acetylcholine (ACh (**A**,**B**)), insulin (**C**,**D**) and diethylamine NONOate (DEA-NO (**E**,**F**)) in aorta segments with perivascular adipose tissue (PVAT+) or without (PVAT-) from control and LysM-GRK2^+/−^ mice fed on normal diet (ND) or high (HFD) fat diet (*n* = 7–19); * *p* < 0.05 vs. PVAT−, # vs. control mice, by two-way ANOVA.

To directly address whether GRK2 dosage in myeloid cells might have a role in vascular functionality and damage, we used a HFD-induced obesity model comparing control animals with those with a selective downregulation of GRK2 in this cell lineage (LysM-GRK2^+/−^). We have recently shown that a 12 week-long HFD feeding induced obesity in both control and LysM-GRK2^+/-^ animals to the same extent but only deteriorated glucose tolerance, caused hyperglycemia, and provoked insulin resistance and inflammation in liver and adipose tissue in control mice, while LysM-GRK2^+/−^ animals maintained smaller adipocytes [23]. Regarding vascular function, we show here that, in aortic rings devoid of PVAT, endothelium-dependent relaxation to acetylcholine was similar in control and LysM-GRK2^+/−^ mice, both in animals fed a normal diet (ND) (Figure 2A) and in animals fed a HFD (Figure 2B). In the presence of PVAT, acetylcholine-induced relaxation was similarly rightward-shifted in arteries from both genotypes when fed a ND (Figure 2A), indicating a lower vasodilator capacity of vessels in the presence of PVAT. However, when fed a HFD, the presence of PVAT impaired acetylcholine-induced relaxation only in arteries from control mice and not in arteries from LysM-GRK2^+/−^ mice (Figure 2B). 

We next evaluated insulin-induced vasodilator responses. Similar to acetylcholine, in aortic segments without PVAT, insulin-dependent relaxation was similar between control and LysM-GRK2^+/−^ mice, both in animals fed a ND (Figure 2C) and a HFD (Figure 2D). In the presence of PVAT, insulin-induced relaxation was significantly impaired in arteries from control animals fed a ND (Figure 2C); moreover, in control animals fed a HFD, insulin did not produce any measurable relaxant response (Figure 2D). Importantly, in arteries from LysM-GRK2^+/−^ mice, the presence of PVAT did not impair insulin responses, neither in animals fed a ND nor in those fed a HFD (Figure 2C,D).

Endothelium-independent relaxations induced by the NO-donor DEA-NO were similar in arteries without PVAT from control and LysM-GRK2^+/−^ mice, both in animals fed with normal (Figure 2E) or HFD (Figure 2F). The presence of PVAT very slightly impaired DEA-NO relaxation in arteries from control mice fed a ND or a HFD but not in vessels from LysM-GRK2^+/−^ mice (Figure 2E,F). These results indicate that reduced levels of GRK2 in myeloid cells prevent the PVAT-induced impairment of vasorelaxation, particularly in arteries from HFD-fed animals.

**Figure 3:**
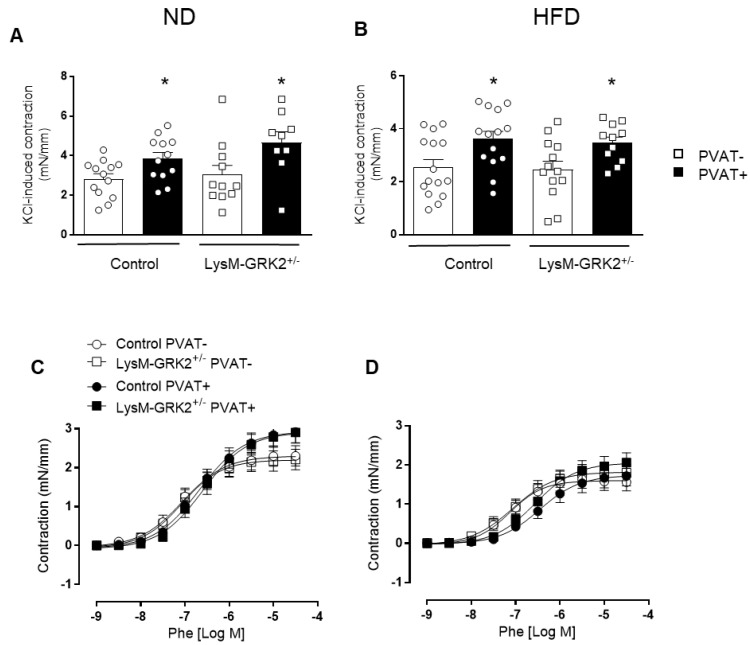
Maximum response induced by 120 mmol/L K^+^ solution (**A**,**B**) and concentration-response curves to phenylephrine (Phe; (**C**,**D**)), in aorta segments with perivascular adipose tissue (PVAT+) or without (PVAT−) from control and LysM-GRK2^+/−^ mice fed on normal (ND) or high (HFD) fat diet (*n* = 9–17); * *p* < 0.05 vs. PVAT− by unpaired t-test (**A**,**B**).

We also evaluated vasoconstrictor responses. As shown in Figure 3, arteries with PVAT showed greater contractile responses induced by KCl independently of the genotype or type of diet (Figure 3A,B). However, no differences in the contractile response to phenylephrine were observed in arteries in the presence or in the absence of PVAT from ND- or HFD-fed animals (Figure 3C,D). Together, these data suggest that GRK2 present in myeloid cells modulates the phenotype of PVAT to release inter-cellular mediators that impair endothelium-dependent relaxations to acetylcholine and insulin but not of contractility towards phenylephrine.

### 3.3. GRK2 Deficiency in Myeloid Cells Prevents Infiltration of Immune Cells and Upregulation of TNFα and Nox1 in PVAT from Obese Animals and Blockade of These Pathways Rescues Vasodilator Responses to Insulin in Arteries with PVAT from HFD-Fed Animals

**Figure 4:**
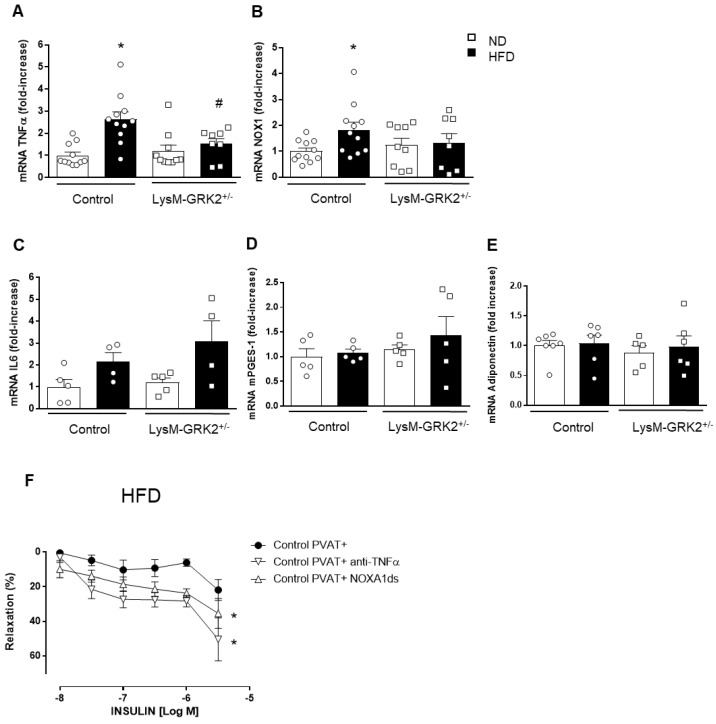
mRNA expression of tumor necrosis factor-α **(**TNFα) (**A**), the NADPH oxidase (Nox) subunit Nox1 (**B**), IL6 (**C**), microsomal prostaglandin E synthase (mPGES-1 (**D**) and adiponectin (**E**) in aortic perivascular adipose tissue from control and LysM-GRK2^+/−^ mice fed on normal (ND) or high (HFD) fat diet. * *p* < 0.05 vs. ND, # vs. control mice by Mann–Whitney or unpaired t-test when normality is reached (by Shapiro–Wilk). (**F**) Concentration-response curves to insulin in aorta segments with perivascular adipose tissue (PVAT+) in the absence or in the presence of an anti-TNFα antibody or the specific Nox1 inhibitor NOXA1ds from control mice fed on high fat diet (HFD) (*n* = 4–5); * *p* < 0.05 vs. arteries in the absence of inhibitors by two-way ANOVA.

We then analyzed possible factors differentially expressed in PVAT that might modulate endothelium-dependent vasodilator responses. As shown in Figure 4, HFD increased gene expression of TNFα and the Nox1 subunit of the NADPH oxidase in PVAT from control but not from LysM-GRK2^+/−^ mice (Figure 4A,B). To test whether the observed alterations in TNFα expression might be caused by myeloid cells themselves or by adipocytes, we used an *in vitro* system in which 3T3L1 adipocytes were treated with the conditioned media from LPS-stimulated control or GRK2^+/−^ macrophages (see Supplementary Materials and Methods). We found that the increased expression of TNFα appears to be due to inflammatory cells and not to adipocytes inside PVAT since the expression of this cytokine in differentiated 3T3L1 adipocytes exposed to conditioned media of LPS-stimulated macrophages is not different in those exposed to the conditioned media of control macrophages compared to those exposed to supernatants from LysM-GRK2^+/−^ macrophages (Figure S1). No differences in the expression of other inflammatory mediators, such as interleukin 6 (IL6), microsomal prostaglandin E synthase 1, nor in the expression of adiponectin were observed between diets or genotypes (Figure 4C–E).

To confirm a possible functional implication of TNFα and Nox1 in PVAT in the impairment of insulin-induced vasodilator responses in arteries with PVAT from control HFD-fed animals, we tested the effect of a TNFα blocking antibody or of the inhibitor of Nox1, NOXA1ds, in insulin-induced vasorelaxation. As shown in Figure 4F, both inhibitors significantly improved the vasodilation response to insulin, which indicates that both TNFα and Nox1 play a role in the negative modulation of vasorelaxation produced by PVAT from HFD-fed animals.

**Figure 5:**
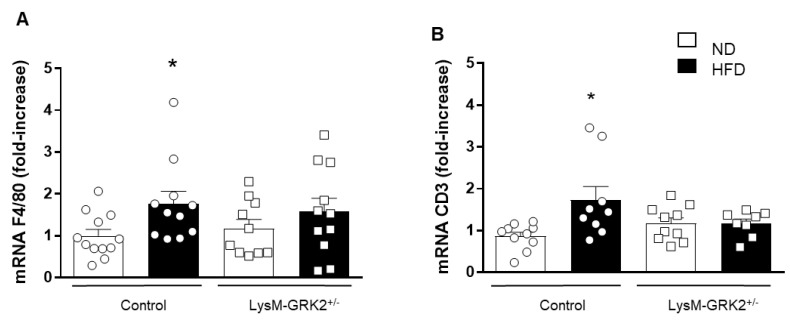
mRNA expression of F4/80 (**A**) and CD3 (**B**) in aortic PVAT from control and LysM-GRK2^+/−^ mice fed a normal (ND) or a high fat diet (HFD). * *p* < 0.05 vs. ND by Mann–Whitney or unpaired *t*-test when normality is reached (by Shapiro–Wilk).

We then looked at the expression of inflammatory markers indicative of cells infiltrated in PVAT that could be involved in TNFα secretion. As shown in Figure 5, a HFD feeding produced a significant increase in the macrophage marker F4/80 and in the T lymphocyte marker CD3 in PVAT of control but not of LysM-GRK2^+/−^ mice.

### 3.4. The Expression of TNFα in PVAT from Patients with Abdominal Aortic Aneurysms Positively Correlates with Obesity 

We then analyzed different inflammatory parameters in PVAT from patients with AAA and their correlation with BMI and abdominal perimeter. As shown in Table 3, we did not find a significant correlation between macrophage or lymphocyte markers with either BMI or abdominal perimeter. However, we found a positive significant correlation between TNFα and BMI and nearly significant with abdominal perimeter. Altogether, these results highlight the association of PVAT-derived TNFα to vascular damage in the context of human obesity.

## 4. Discussion

In this work, we uncovered that, in aortic PVAT from patients with extensive vascular damage, there is a positive and highly significant correlation between GRK2 expression and macrophage and lymphocyte markers. Moreover, we show here that a partial deficiency in GRK2 solely in myeloid cells can prevent the impairment of endothelium-dependent vasodilator responses induced by PVAT in a model of HFD, in the absence of significant effects on vasoconstriction. This effect appears to involve a decreased capacity for the HFD to infiltrate macrophages and T lymphocytes and to upregulate Nox1 and TNFα in PVAT from LysM-GRK2^+/−^ mice.

PVAT is a secretory organ involved in the regulation of hemodynamic homeostasis, and a balance between PVAT-derived vasodilator and vasoconstrictor mediators appears to be important to maintain an appropriate vascular tone [3,4]. Even when the volume of PVAT is associated with hypertension and aortic and coronary calcification, it is the function of PVAT, rather than the size of this tissue, which seems to be key for the control of vascular homeostasis [5]. Since PVAT is highly adaptable to changes in environmental stimuli, such as a HFD, it more readily promotes a pro-inflammatory state compared with other adipose depots. For instance, mice fed a HFD reduce the expression of anti-inflammatory and increase that of pro-inflammatory adipokines and cytokines in PVAT from the aortic arch in the first two weeks of HFD feeding, a period in which only minor changes are detected in visceral and subcutaneous fat [25]. Increased infiltration of immune cells, including macrophages and T lymphocytes, is a hallmark of PVAT dysfunction not only in obesity but also in other cardiovascular diseases, such as abdominal aortic aneurysms, and both cell types play an important role in vascular alterations associated with these pathologies, in part through the modulation of vasoactive responses (reviewed in Reference [8,24]). Here, we find that PVAT impairs endothelium-dependent vasodilator effects to acetylcholine and insulin in animals fed a ND or a HFD, which confirms the ability of PVAT to modulate vascular responses. Notably, in animals fed a HFD, segments with PVAT were unable to achieve any observable relaxant response to insulin. Because NO-independent relaxation is only minimally affected by the presence of PVAT, the observed effects induced by PVAT could probably be due to a decrease in NO availability. 

Early studies from our group demonstrated that GRK2 is involved in the regulation of whole organism glucose homeostasis and also of local insulin resistance in different tissues (reviewed in Reference [16,26]). Here, we find that, in the absence of PVAT, endothelium-dependent or -independent vasodilator responses are unaltered by GRK2 downregulation in myeloid cells either in mice fed a ND or a HFD. However, the PVAT-induced impairment of acetylcholine or insulin responses is completely prevented by reducing GRK2 in myeloid cells in animals fed a HFD. This uncovers a new mechanism of regulating the development of endothelial dysfunction, and vascular insulin resistance induced by PVAT in the context of obesity. Of note, we cannot rule out that an insulin-resistant PVAT may impact signaling pathways inside the endothelium (or inside vascular smooth cells) to alter vascular reactivity. Interestingly, vascular contractile responses were unaffected by the presence of PVAT or by the genotype of the animals. The beneficial effects of GRK2 reduction are likely due to an increase in NO availability rather than to altered vascular smooth muscle NO sensitivity, since DEA-NO-induced responses were unaffected by the genotype. Coherently, the increased expression of GRK2 observed in vessels from different mouse models of vascular or metabolic diseases correlates with a decrease in NO bioavailability that may contribute to endothelial dysfunction [26,27]. In C57Bl6/J mice, in particular, lowering GRK2 increases NO bioavailability in vessels [17]. Whether this might also affect vascular remodeling or blood pressure in our model remains an attractive hypothesis that deserve further investigation. In any case, the particular effect of decreased GRK2 in myeloid in vascular biology was not addressed in prior studies. 

The positive correlation between GRK2 levels and macrophage- and lymphocyte-specific markers in PVAT of patients with abdominal aortic aneurysms highlights the importance of GRK2 in immune cells in this specific adipose tissue depot. In our control mice, a 12-week-long HFD is able to increase the expression in PVAT of one of the most important PVAT-derived cytokines, TNFα. However, LysM-GRK2^+/−^ mice appear to be protected from this HFD-induced TNFα upregulation. Notably, we find that, in PVAT from patients with AAA, there is a significant correlation between TNFα and BMI and nearly significant with abdominal perimeter. It has been suggested that, during a HFD, PVAT inflammation precedes macrophage infiltration. So, the recruitment of macrophages seems to occur in response to PVAT inflammation [25]. Infiltrating macrophages may, in turn, potentiate the inflammatory response of PVAT and enhance the activity of NADPH oxidase [28], further enhancing PVAT inflammation in a positive feedback loop. Our results suggest that lowering GRK2 in myeloid cells appears to have an impact not only in macrophages but also in T lymphocytes, thus affecting the infiltration of different populations of immune cells in the PVAT. This might contribute to decrease TNFα expression and to deteriorate less endothelial function. Whether this decreased recruitment is due to a reduced inflammatory response of PVAT at an early stage during the HFD feeding or rather is a consequence of the reduced levels of GRK2 within macrophages deserves further investigation. Our data on 3T3L1 cells also suggest that macrophages are the main producers of TNFα, even when we cannot fully discard a possible participation *in vivo* of adipocytes and/or T cells inside PVAT to the differential phenotype observed. Interestingly, the results we describe here resemble the phenotype observed in other adipose depots from this same mouse strain [23]. In particular, TNFα expression in the visceral white adipose tissue was decreased in HFD-fed LysM-GRK2^+/−^ mice due to a reduced amount of the M1 type of pro-inflammatory macrophages infiltrating this adipose depot [23]. Moreover, GRK2 levels in human PVAT correlate with that of pro-inflammatory adipokines, such as leptin, in agreement with data obtained in visceral adipose depots of LysM-GRK2^+/−^ animals fed a HFD where leptin levels were decreased when GRK2 amount was reduced [23]. On the contrary, GRK2 mRNA did not correlate with adiponectin in human PVAT in agreement with the lack of differences in adiponectin levels in murine PVAT between the two genotypes. Given the described pro-inflammatory effects of leptin and its implication in the increased cardiovascular risk observed in obese patients [29], these data support an additional mechanism associating increased GRK2 levels with enhanced inflammation and vascular dysfunction by means of increasing PVAT-derived leptin production. Altogether, these results suggest that low levels of GRK2 in myeloid cells can keep at bay the pro-inflammatory reprogramming taking place in different adipose depots during diet-induced obesity. 

Since macrophage activation plays a key role in mediating the vascular actions of PVAT, and the presence of macrophages in this tissue is required for PVAT-mediated changes in artery contractility following inflammation [30], this reduced pro-inflammatory profile in the PVAT from LysM-GRK2^+/−^ mice may be able to blunt the vascular deterioration observed in this pathological setting. This is demonstrated by the preserved vasodilation towards insulin and acetylcholine observed in these mice. In fact, the key importance of TNFα in vascular tone is demonstrated by studies showing that a direct application of TNFα to the PVAT around healthy blood vessels decreases PVAT-induced beneficial effects on the vasculature [9]. Importantly, high levels of free fatty acids in rat aorta have been suggested to induce TNFα upregulation and inflammation in the PVAT depot, and to attenuate its anti-contractile properties [31]. The loss of the beneficial effects of PVAT in obese humans [9] or in mice in inflammatory conditions [30] can be rescued not only by anti-TNFα antibodies but also by catalase and superoxide dismutase, which highlights the key importance of reactive oxygen species (ROS) in the PVAT-mediated control of vascular function. In fact, in obese individuals, the upregulation of superoxide anion and ROS may feed forward on the reduction of endothelial NO production and vasorelaxant actions [4,6]. Moreover, an increased production of ROS leading to a loss of the anti-contractile effect of thoracic PVAT was demonstrated in a different murine model [32], and enhanced NADPH oxidase and superoxide anion production has been found in thoracic PVAT of mice after an 8-week-long HFD (60% Kcal from fat), which results in endothelial dysfunction [33]. PVAT is essential for this effect since removal of this tissue significantly improved endothelium-dependent relaxation. Consistent with all these reports, we detect an increase in the expression of the Nox1 subunit of NADPH oxidase in the PVAT of obese control mice that does not occur in LysM-GRK2^+/−^ animals. Additionally, in the cardiomyocyte cell line H9c2 overexpression of GRK2 is enough to trigger ROS production in a NADPH oxidase-dependent manner, and this kinase also seems to be required for the adrenergic-mediated stimulation of ROS production [34]. Even when the precise mechanism by which GRK2 may directly or indirectly activate NADPH oxidase is still missing, altogether, these studies provide a link between GRK2 levels and NADPH activity or expression that deserves further investigation.

**Figure 6:**
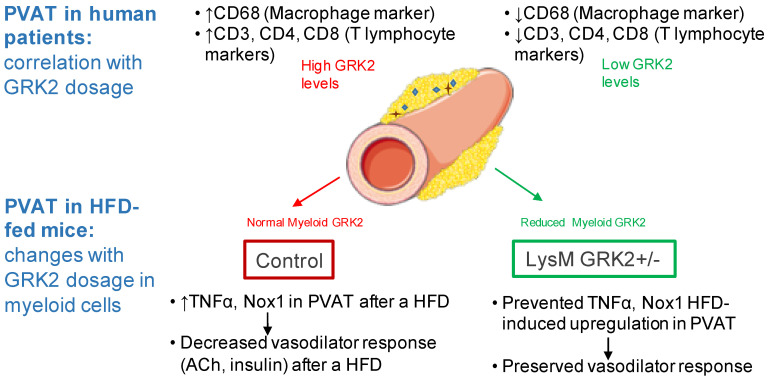
Schematic representation of the correlation of GRK2 levels with inflammatory markers in human PVAT and of the effects of downregulating GRK2 in myeloid cells for the vascular responses in vessels with PVAT after the induction of obesity.

Although our experimental set up does not allow us to precisely differentiate whether ROS and TNFα are interrelated mechanisms, TNFα is capable of generating an excess of ROS via NADPH oxidase activation and also induces endothelial NO synthase (eNOS) uncoupling, which generates superoxide anions, thus impairing NO bioavailability and endothelium-dependent relaxation [6]. In fact, inflammation and oxidative stress are intertwined processes associated with adipose tissue and vascular dysfunction [35], and the relationship between both processes comes from the fact that macrophages represent a key effector of both the production of ROS and cytokines [36]. Coherently, we demonstrate here that both TNFα-dependent effects and Nox1-derived ROS are responsible for the inhibitory effect of PVAT on insulin responses in animals after a HFD feeding. And we observe that, in mice with decreased levels of GRK2 in macrophages, a HFD fails to hamper vascular function in the face of this nutrient overload and in a PVAT-dependent manner. Thus, as summarized in Figure 6, we propose that the beneficial effect of GRK2 downmodulation in myeloid cells in maintaining vasodilator responses derives from limiting the levels of both inflammatory and oxidative mediators in PVAT.

## 5. Conclusions

In sum, our results suggest that GRK2 in myeloid cells may play an important role in orchestrating the responses that promote both inflammation and excessive ROS production by NADPH oxidase in PVAT to decrease endothelium-dependent vasodilation. Lowering myeloid GRK2 protein could prevent this vicious cycle from establishing and thus stop vascular dysfunction by preventing, on the one hand, the pro-inflammatory shift and, on the other, the increased NADPH oxidase expression observed during obesity. Altogether, these results uncover a potentially novel strategy for the regulation of vascular dysfunction in obesity that may have therapeutic implications for the treatment of vascular disorders.

## Figures and Tables

**Table 1:** Patient characteristics.

Clinical and Laboratory Parameters	
Gender (M/F)	39/3
Age (Years)	70.32 ± 1.122
Body weight (Kg)	85.8 ± 2.257
Height	1.711 ± 0.011
BMI (kg/m²)	29.31 ± 0.756
Abdominal perimeter (cm)	109 ± 2.01
Smoking (no/yes/ex)	4/15/24
Diabetes mellitus (yes/total)	10/42
Hypertension (yes/total)	29/42
Hyperlipidemia (yes/total)	26/42
* Cardiopathies (yes/total)	19/42
CKD	12/42
COPD	13/42
Medication	
Antihypertensive	29/39 (74%)
Lipid lowering drugs	30/39 (77%)
Antidiabetic	9/39 (23%)
Antiaggregant	22/39 (56%)
Anticoagulant	7/39 (18%)
Beta blockers	14/39 (36%)

Values are expressed as mean ± SEM, number of subjects or percentages. * Cardiopathies include congenital heart disease, ischemic cardiomyopathy, valvular cardiopathy, heart failure, arrhythmia, dilated cardiomyopathy, open-heart surgery and previous coronary endovascular procedure. CKD: Chronic Kidney Disease, COPD: Chronic Obstructive Pulmonary Disease.

**Table 2:** Real-time PCR primes sequences or Taqman probes ID.

GENE NAME	NCBI Seq	Forward Sequence	Reverse Sequence
Mice			
*Tnfa*	NM_013693	5′CCACGCTCTTCTGTCTACTG	5′TGAGGGTCTGGGCCATAGA
*Nox1*	NM_172203	5′CAACAGCACTCACCAATGCC	5′ACATCCTCACTGACTGTGCC
*Il6*	NM_031168	5′TGATGGATGCTACCAAACTGG	5′TTCATGTACTCCAGGTAGCTATGG
*Ptges*	NM_022415	5′AGGATGCGCTGAAACGTGGAG	5′CCGAGGAAGAGGAAAGGATAG
*Adipoq*	NM_009605	5′TGATGGCAGAGATGGCACTC	5′CTGTCTCACCCTTAGGACCA
*Adgre1 (F4/80)*	NM_001256252	5′GTTCAGGGCAAACGTCTCG	5′TGCTCTAACTCTGTGGGAAGC
*Cd3*	NM_007648	5′TATGGCTACTGCTGTCAGGT	5′TGGCTACTACGTCTGCTACA
*B2m*	NM_009735	5′ACCCTGGTCTTTCTGGTGCTT	5′TAGCAGTTCAGTATGTTCGGCTT
Human			
*ACTB* *(β-actin)*	NM_009735	5′-AGAGCTACGAGCTGCCTGAC	5′-AGCACTGTGTTGGCGTACAG
*CD68*	NM_001101	5′-TAGCTGGACTTTGGGTGAGG	5′-CCAGTGCTCTCTGCCAGTA
*CD3*	NM_001040059	5′-TCTACCAGCCCCTCAAGGAT	5′-AGGAGGAGAACACCTGGACTA
*CD4*	NM_000073	Hs00181217	
*CD8a*	NM_000616.4	Hs00233520	
*GRK2*	NM_001145873	Hs00176395_m1	
*TNFa*	NM_001619	Hs.PT.58.45380900	
*NOX5*	NM_024505	Hs00225846_m1	
*RNA18S1*	106632259	4310893E	

**Table 3:** Correlation between body mass index (BMI) or abdominal perimeter (Ab. perimeter) with different immune infiltration markers.

Parameter	BMI	Ab. Perimeter
BMI	-	*p* = 0.001 *
Ab. Perimeter	*p* = 0.001 *	-
CD68	*p* = 0.33	*p* = 0.57
CD3	*p* = 0.41	*p* = 0.32
CD4	*p* = 0.46	*p* = 0.07
CD8	*p* = 0.53	*p* = 0.24
TNFα	*p* = 0.02 *	*p* = 0.06

The correlation between BMI or abdominal perimeter with the mRNA of the macrophage marker CD68, the T lymphocyte markers CD3, CD4, and CD8, and TNFα mRNA is analyzed in human aortic PVAT. Univariate association was performed by Spearman correlation test. * *p* < 0.05.

## Supplementary Materials

The following are available online at https://www.mdpi.com/2076-3921/9/10/953/s1, Figure S1: mRNA expression of TNFα and IL6 in 3T3L1 adipocytes, Table S1: Primers used for real time PCR analysis in 3T3L1 adipocytes.
