# Supplementary material for: Myeloid GRK2 Regulates Obesity-Induced Endothelial Dysfunction by Modulating Inflammatory Responses in Perivascular Adipose Tissue
Source: Antioxidants (Basel). 2020 Oct 4;9(10):953. doi: 10.3390/antiox9100953 (PMC7600489; doi:10.3390/antiox9100953)
Supplement: Supplementary file 1 [file antioxidants-09-00953-s001.pdf]

antioxidants MDPI

TITLE

Myeloid GRK2 regulates obesity-induced endothelial dysfunction by modulating inflammatory responses in perivascular adipose tissue.

Authors
[1,2]María González-Amor, [3,+]Rocío Vila-Bedmar, [1,2,+]Raquel Rodrigues-Díez, [4]Rosa Moreno-Carriles, [2,5,6]Alba C. Arcones, [2,5,6]Marta Cruces-Sande, [1,2]Mercedes Salaices, [2,5,6]Federico Mayor Jr., [1,2*]Ana M. Briones and [2,5,6,*]Cristina Murga

[1]Dpt. Farmacología, Facultad de Medicina, Universidad Autónoma de Madrid, Instituto de Investigación Hospital La Paz, Madrid, Spain.
[2]Ciber de Enfermedades Cardiovasculares (CIBERCV), Spain.
[3]Dpt de Ciencias Básicas de la Salud, Facultad de Ciencias de la Salud, Universidad Rey Juan Carlos (URJC), Spain.
[4]Servicio de Angiología y Cirugía Vascular. Hospital Universitario La Princesa. Madrid.
[5]Departamento de Biología Molecular and Centro de Biología Molecular Severo Ochoa (CBMSO) UAM-CSIC, Madrid, Spain.
[6]Instituto de Investigación Sanitaria Hospital Universitario La Princesa, Madrid, Spain.
[+]These authors contributed equally to this work.
[*] Correspondence: cristina.murga@uam.es (C.M.); ana.briones@uam.es (A.M.B.)

Supplementary material

Supplementary Figure 1.

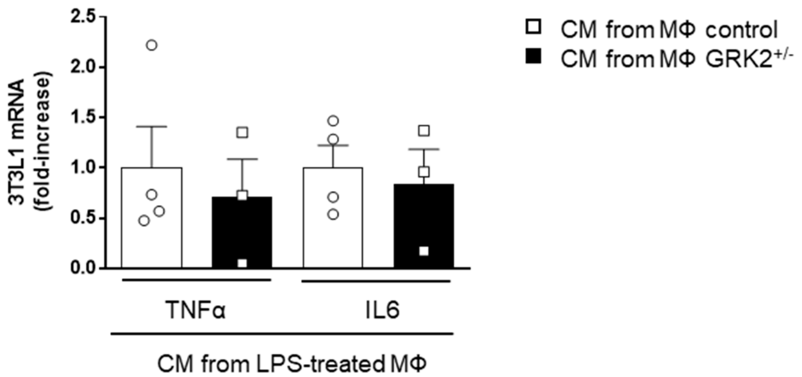

Legend to Supplementary Fig. 1. *mRNA expression of TNFα and IL6 in 3T3L1 adipocytes stimulated for 24h with the conditioned medium (CM) obtained from macrophages (MΦ) isolated from either control or LysM-GRK2*$^{+/-}$ *mice; these macrophages were previously treated with 1µg/mL LPS for 6 hours. Graphs represent fold change over control (3T3L1 adipocytes stimulated with the CM of LPS-treated control macrophages). Data are expressed as mean ± SEM. Statistical significance was analyzed by two-way ANOVA followed by Bonferroni posthoc test (n=3-4).*

Supplementary Methods

3T3L1 preadipocytes were cultured in DMEM supplemented with 10% FBS and differentiated as previously described (Vila-Bedmar et al., *FASEB J* 2012 26(8):3503). At day 10 post-differentiation, adipocytes were serum starved (1% FBS) overnight and further incubated with the conditioned medium (CM) of thioglycollate elicited peritoneal macrophages obtained from control or LysM-GRK2$^{+/-}$ mice, treated or not with 1 μg/mL LPS for 6 hours. 24h later, 3T3L1 adipocytes were lysed with QIAzol reagent (Qiagen) to isolate RNA. Quantity and quality of RNA were analyzed using Nanodrop ND-1000 (Thermo Scientific) and Bioanalyzer 2100 (Agilent). RT-PCRs were performed with the aid of the Genomic Facility of the Centro de Biología Molecular Severo Ochoa using Light Cycler equipment (Roche) with Sybr Green technology and self-designed murine probes purchased from Sigma-Aldrich (Supplementary Table 1). *Ppia* was used as reference gene following the recommendations of GenNorm and NormFinder algorithms.

Supplementary Table 1. Primers used for real time PCR analysis in 3T3L1 adipocytes:

GENE NAME	NCBI seq	Forward sequence	Reverse sequence
Mice			
Tnfa	NM_013693	5' TCTTCTCATTCCTGCTTGTGG	5' GGTCTGGGCCATAGAACTGA
Il6	NM_031168	5'TGATGGATGCTACCAAACTGG	5'TTCATGTACTCCAGGTAGCTATGG
Ppia	NM_008907	5'ACGCCACTGTCGCTTTTC	5'GCAAACAGCTCGAAGGAGAC

References

GRK2 contribution to the regulation of energy expenditure and brown fat function. Vila-Bedmar R, Garcia-Guerra L, Nieto-Vazquez I, Mayor F Jr, Lorenzo M, Murga C, Fernández-Veledo S. FASEB J. 2012 Aug;26(8):3503-14. doi: 10.1096/fj.11-202267.
